# Immune response in dairy cattle against combined foot and mouth disease and haemorrhagic septicemia vaccine under field conditions

**DOI:** 10.1186/s12917-021-02889-8

**Published:** 2021-05-05

**Authors:** Anucha Muenthaisong, Amarin Rittipornlertrak, Boondarika Nambooppha, Pallop Tankaew, Thanya Varinrak, Marutpong Pumpuang, Korkiat Muangthai, Kheemchompu Atthikanyaphak, Tawatchai Singhla, Kidsadagon Pringproa, Veerasak Punyapornwithaya, Takuo Sawada, Nattawooti Sthitmatee

**Affiliations:** 1 Department of Veterinary Bioscience and Veterinary Public Health, Faculty of Veterinary Medicine, Chiang Mai University, 50100 Chiang Mai, Thailand; 2 Central Laboratory, Faculty of Veterinary Medicine, Chiang Mai University, 50100 Chiang Mai, Thailand; 3 Bureau of Veterinary Biologics, Department of Livestock Developments, Ministry of Agriculture and Cooperative, 30130 Nakhon Ratchasima, Thailand; 4 Laboratory of Veterinary Microbiology, Nippon Veterinary and Life Science University, 180-8602 Musashino, Tokyo, Japan; 5 Excellence Center in Veterinary Bioscience, Chiang Mai University, 50100 Chiang Mai, Thailand

**Keywords:** Combination vaccine, Dairy cattle, Foot and Mouth disease, *Pasteurella multocida*, rOmpH

## Abstract

**Background:**

Foot-and-mouth disease (FMD) and Haemorrhagic septicemia (HS) are two important diseases that are known to have caused significant economic losses to the cattle industry. Accordingly, vaccinations have been recognized as an efficient method to control and prevent both of the above-mentioned diseases. This study aimed to determine the immune response to FMD virus antigens and the recombinant outer membrane protein of HS (rOmpH) of *Pasteurella multocida* in cattle administered as a combination vaccine and compare antibody titers with the two vaccines given independently, under field conditions. Dairy cattle were divided into three groups. Each group was immunized with different vaccine types according to the vaccination program employed in this study. Antibody responses were determined by indirect ELISA, liquid phase blocking ELISA (LPB-ELISA) and viral neutralization test (VNT). Furthermore, the cellular immune responses were measured by lymphocyte proliferation assay (LPA).

**Results:**

The overall antibody titers to HS and FMDV were above cut-off values for the combined FMD-HS vaccine in this study.The mean antibody titer against HS after the first immunization in the combined FMD-HS vaccine groups was higher than in the HS vaccine groups. However, no statistically significant differences (*p* > 0.05) were observed between groups. Likewise, the antibody titer to the FMDV serotypes O/TAI/189/87 and Asia 1/TAI/85 determined by LPB-ELISA in the combined vaccine were not statistically significantly different when compared to the FMD vaccine groups. However, the mean VNT antibody titer of combined vaccine against serotype O was significantly higher than the VN titer of FMD vaccine groups (*p* < 0.05). Moreover, the LPA results showed that all vaccinated groups displayed significantly higher than the negative control (*p* < 0.05). Nevertheless, no differences in the lymphocyte responses were observed in comparisons between the groups (*p* > 0.05).

**Conclusions:**

The combined FMD-HS vaccine formulated in this study could result in high both antibody and cellular immune responses without antigenic competition. Therefore, the combined FMD-HS vaccine can serve as an alternative vaccine against both HS and FMD in dairy cattle under field conditions.

## Background

The outbreak of an infectious disease that is caused by either pathogenic bacteria or viruses can lead to significant losses in the industrial production of animal-based products worldwide. Foot and mouth disease (FMD) is one of the most highly contagious viral diseases of cloven-hoofed animals. It has caused severe economic losses of between US$6.5 and 21 billion [1, 2]. Furthermore, hemorrhagic septicemia (HS) caused by *P. multocida* has been reported to have caused economic losses of US$ 792 million per year in the livestock industry in India’s livestock sector [3–5]. Consequently, both the prevention of infectious diseases and issues related to animal welfare have become significant concerns since the raising of cattle on an industrial scale is a major component of the broader cattle industry.

The administration of vaccinations is considered an efficient strategy in the control of diseases among cattle. Vaccinations appear to have been the only practical approach in the prevention of HS disease [6–8]. Various formulations of HS vaccines are available including live vaccines, inactivated vaccines, purified capsular extract vaccines and combined vaccines [9]. The outer membrane protein H (OmpH), a major membrane protein located on the envelope and capsule of *P. multocida*, has displayed a strong potential for immunogenicity [10–12]. In the last few decades, OmpH has been recognized as a vaccine with significant potential against several diseases caused by *P. multocida* such as fowl cholera in chickens [13–16] and ducks [17], shipping fever [18] and swine atrophic rhinitis [19]. In our study, the OmpH molecular mass was found to vary from 32 to 39 kDa [20]. Notably, 37 kDa of OmpH was found to be the major immunogenic protein of the *P. multocida* serotype B:2, which is known to cause HS in cattle and buffaloes [21, 22]. In our research study, we were able to confer a strong antibody titer and an effective degree of protective immunity against *P. multocida* infection in mice models [23]. Furthermore, previous studies have demonstrated that recombinant OmpH (rOmpH) provided protective immunity against *P. multocida* in both cattle and buffaloes [6, 9, 24].

One of the most important pathogenic viruses among cattle is the FMD virus (FMDV). It is a highly contagious acute vesicular viral disease that affects cloven-hoofed animals and is mainly controlled by vaccination. The FMD vaccination is one of the most important tools that can be employed to protect susceptible animals from FMDV infection in endemic countries [25]. However, inactivated vaccines are commonly used to immunize cattle and cloven-hoofed animals worldwide [26]. Nevertheless, boosters are required at intervals of approximately 4–6 months in order to provide full protection [27].

With regard to administering vaccinations in the livestock industry, cost-benefit analyses are very important [28]. Consequently, a combined vaccine would be extremely beneficial to this industry. It would not only help to reduce the cost of developing vaccinations, but it could also help to expand the coverage of administering the vaccine [29]. In Thailand, the administration of FMD vaccine and HS vaccines are routinely conducted among ruminant animals with different vaccination regimes. It would be of significant interest to develop an attractive vaccine against FMDV and HS that could be administered within the livestock industry in a single dose. Several previous studies have revealed that the development of a combined vaccine based on FMD vaccines has shown potential for success. In these studies, the combined vaccine was determined to be safe, well-tolerated and immunogenic [30–32]. Therefore, the present study was aimed to develop a combined FMD-HS vaccine by employing an inactivated FMD vaccine and rOmpH of the *P. multocida* B:2 strain M1404. The subsequent objective would then be to evaluate the combined vaccine’s degree of immunogenicity among dairy cattle under field conditions.

## Results

### Monitoring of antibody titer against heat extract antigen of *P. multocida *strain M-1404 in cattle immunized with rOmpH-containing vaccine formulations

**Fig. 1:**
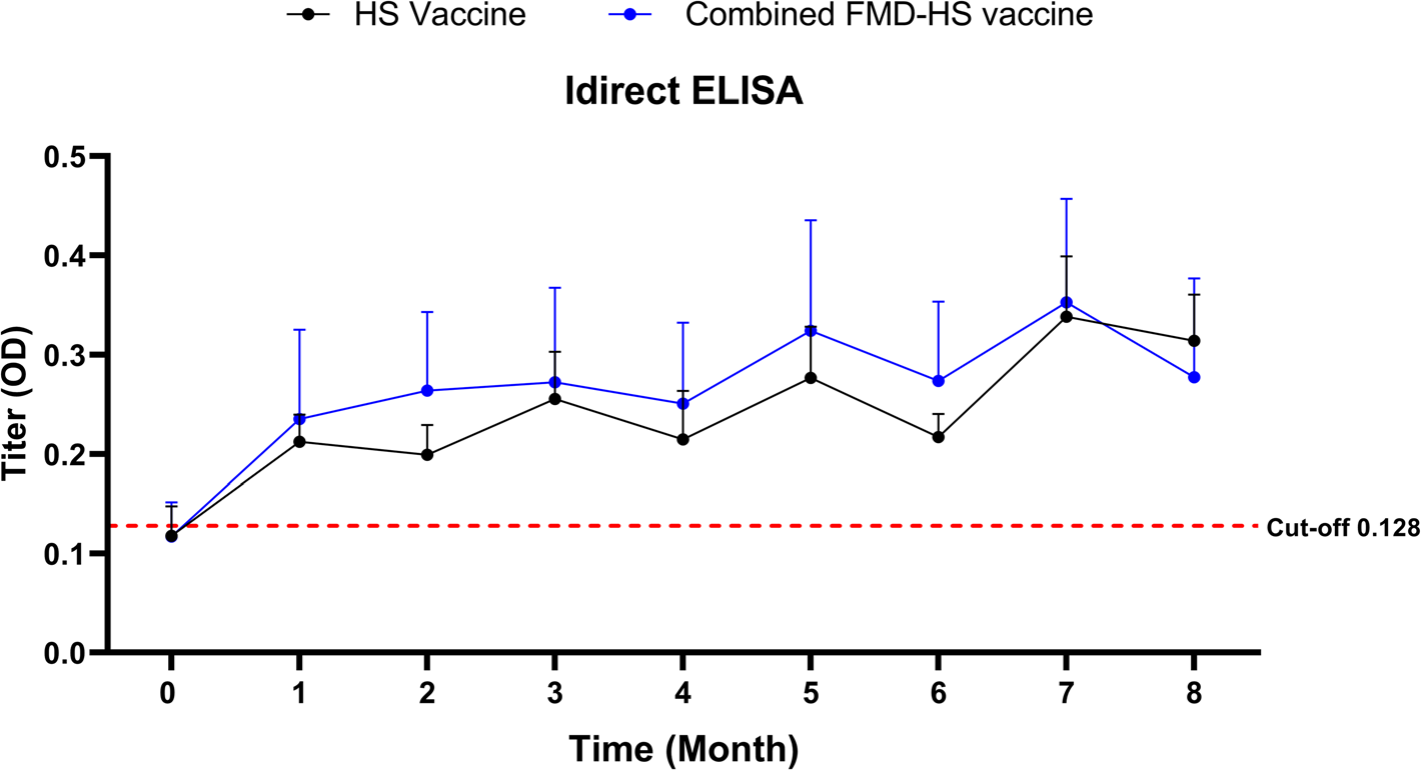
Sera antibody titer levels measured by indirect ELISA against *P. multocida* strain M-1404 unvaccinated and post-vaccination. A values of *p* < 0.05 indicated statistically significant differences

The antibody titer of cattle immunized with rOmpH-containing vaccine formulations is shown in Fig. 1. All cattle were seronegative prior to being vaccinated (the HS group; 0.118 ± 0.028 and the combined FMD-HS vaccine groups; 0.117 ± 0.034). The average antibody levels of the HS and combined FMD-HS groups were higher than the cut-off value (Optical density (OD) = 0.128) after 1-month post-vaccination (MPV). The average FMD-HS vaccine groups (0.281 ± 0.090) was higher than the HS groups (0.254 ± 0.041). However, no significant differences were observed over the course of this investigation (*p* > 0.05).

### Determination of antibody response in cattle immunized with combined FMD-HS vaccine compared to the FMD vaccine

**Fig. 2:**
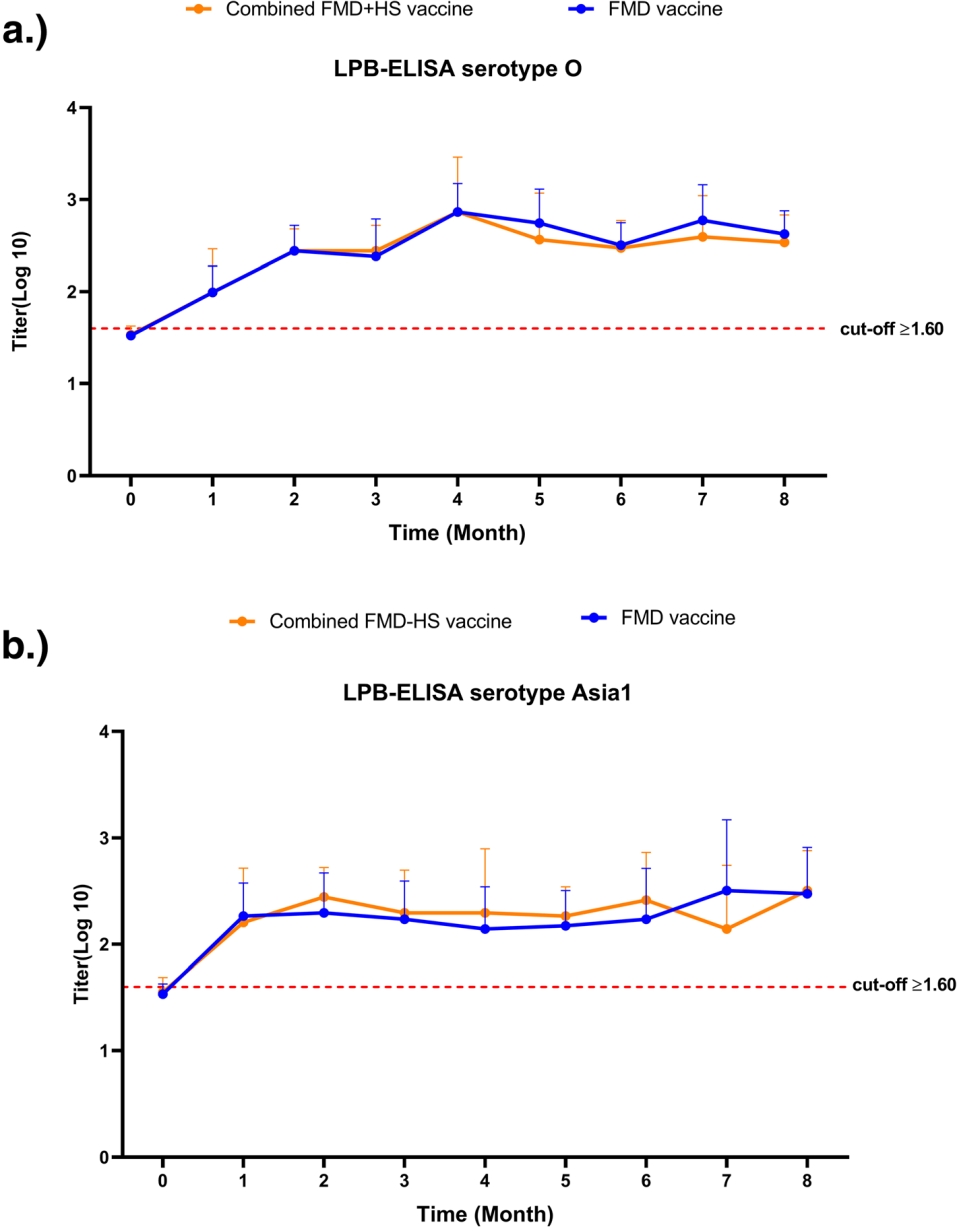
Sera antibody titer levels measured by LPB-ELISA against FMDV serotypes O (**a**) and Asia1 (**b**) unvaccinated and post-vaccination. A value of *p* < 0.05 indicated a statistically significant difference

Liquid phase blocking enzyme-linked immunosorbent assay (LPB-ELISA) revealed antibody responses against the FMDV serotypes O/TAI/189/87 and Asia1/TAI/85 in cattle under field conditions as shown in Fig. 2. The unvaccinated cattle showed antibody titer to FMDV under a cut-off value of 1.6 Log10. The antibody titer of the unvaccinated cattle against FMDV serotype O and Asia1 were 1.562 ± 0.095 and 1.552 ± 0.135, in the combined FMD-HS vaccine group. The antibody titer of the unvaccinated cattle in the FMD vaccine groups were 1.502 ± 0.042 and 1.532 ± 0.095 against FMDV serotype O and Asia1, respectively. Interestingly, the cattle immunized with the FMD-HS combined vaccine group ((2.477 ± 0.374), (2.320 ± 0.436)) demonstrated average anti-FMDV titer to serotypes O/TAI/189/87 and Asia 1/TAI/85 with no significant differences when compared with the FMD vaccine group ((2.540 ± 0.317), (2.290 ± 0.419)) after 1 MPV (*p* > 0.05). Additionally, average levels of the sera antibody obtained from those groups were higher than for the cut-off value for both the FMDV serotypes O and Asia1.

### Neutralizing antibodies derived from combined FMD-HS vaccine against FMDV infection

**Fig. 3:**
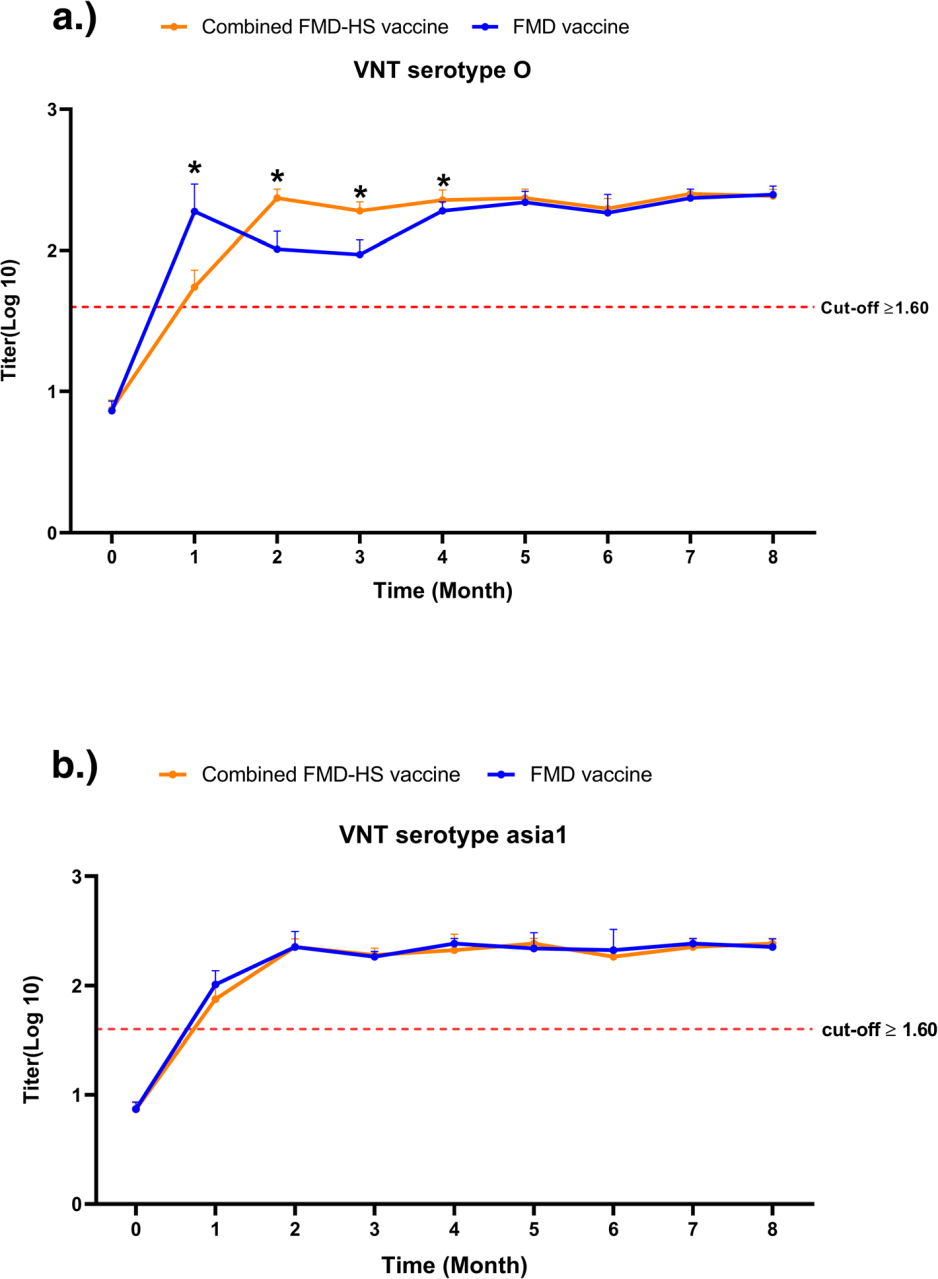
Neutralizing antibody titers measured by VNT against FMDV serotypes O (**a**) and Asia1 (**b**) unvaccinated and post-vaccination. Asterisk (∗) represents the value of *p* < 0.05 indicated a statistically significant difference

The ability of the sera to neutralize the FMDV serotypes O/ TAI/189/87 and Asia 1/TAI/85 is shown in Fig. 3 a and b, respectively. The unvaccinated sera showed lower viral neutralization test (VNT) titers against serotypes O and Asia1 than the cut-off value. The antibody titer of the unvaccinated cattle against the FMDV serotype O in the combined FMD-HS vaccine groups and the FMD vaccine groups were 0.924 ± 0.102 and 0.925 ± 0.091 respectively. Moreover, the VNT antibody titer of the unvaccinated cattle against the FMDV serotype Asia1 were 0.927 ± 0.062 and 0.959 ± 0.091 in the combined HS-FMD vaccine groups and FMD vaccine groups. The average VNT antibody titer of the combined FMD-HS vaccine groups (2.276 ± 0.061) against FMDV serotype O was significantly higher than the FMD vaccine groups (2.085 ± 0.061) (*p* < 0.05) over the course of experiment. Definitely, the significant differences were observed at the first four months of the experiment (*p* < 0.05). However, the mean VNT titer against FMDV serotype Asia1 of combined FMD-HS vaccine groups (2.295 ± 0.080) was not significantly different when compared with the FMD vaccine groups (2.315 ± 0.102) throughout the experiment (*p* > 0.05). However, the mean VNT titers of both the FMD vaccine groups and the combined FMD-HS vaccine groups were higher than the cut-off value as has been determined by OIE.

### Determination of cellular immune response

**Fig. 4:**
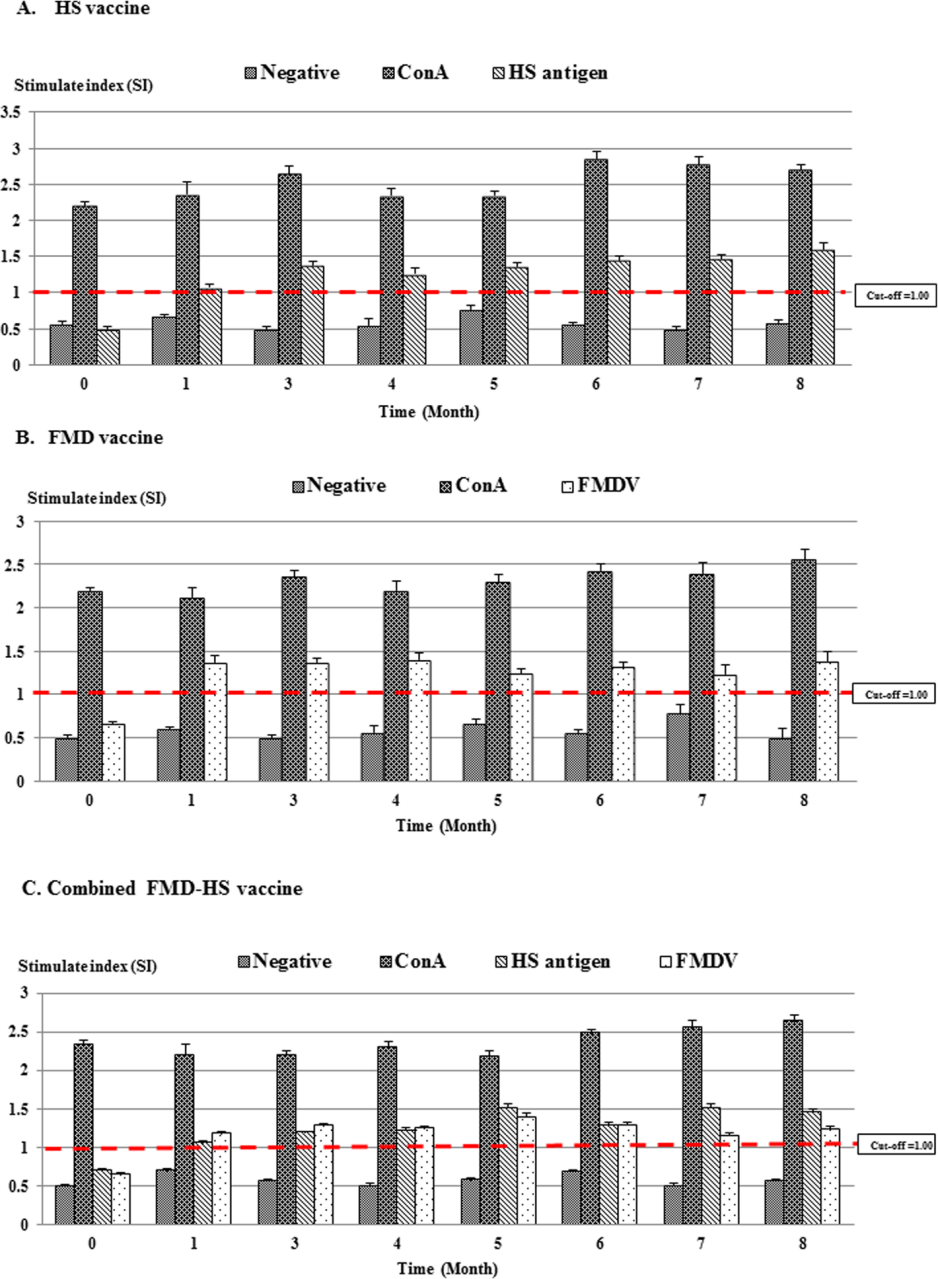
*In vitro* proliferations of lymphocytes in response to FMDV and HS antigens. Results are expressed as stimulation indices. A value of *p* < 0.05 indicated a statistically significant difference

Lymphocyte responses from immunized cattle are shown in Fig. 4. The cellular immune responses against different antigens among unvaccinated cattle were found to be lower than the cut-off value (Stimulation index (SI) = 1 unit). All vaccination groups showed a high degree of SI to ConA stimulation throughout the course of this study. Lymphocyte responses against different antigens indicated that the SI values were increasingly higher than the cut-off value after the first immunization when compared to the non-stimulated control group (*p* < 0.05). No differences in the lymphocyte responses were observed in comparisons between the groups that got the vaccine alone and the combined group (*p >* 0.05*)*.

## Discussion

Prophylactic vaccinations are considered the only practical approach to preventing and controlling FMD and HS among susceptible animals in endemic countries [1, 4, 8]. Consequently, vaccinations against FMDV and HS are routinely practiced in endemic areas [1, 4]. A novel FMD-HS combined vaccine would offer benefits to animals by reducing the number of vaccine doses needed and by increasing the protective immunity of animals against multiple infections with just a single dose. Moreover, it would provide cost-related benefits to the livestock industry. However, interference between combined immunogens could be one of the potential obstacles for the development of a combined vaccine [33]. Accordingly, a previous study has shown that the levels of the FMD titers were lower than normal when animals were simultaneously vaccinated with the two vaccines [34]. However, another study reported that the combination vaccine resulted in no disturbance between the antigens of the combination vaccine. Moreover, the combination vaccine was found to produce a prolonged and stable immune response [29–31].

In the present study, the inactivated bivalent FMD vaccine (alum/saponin) manufactured by the Department of Livestock Development (DLD), Thailand was formulated with rOmpH to produce the FMD-HS combined vaccine against FMD and HS. Immunologically, it is well known that inactivated FMD vaccines focus on humoral immune responses rather than cellular immune responses via the induction of neutralizing antibodies by activating CD4 T-cells [35, 36]. In addition, the alum-based adjuvant could enhance the humoral response through IL-4 by activating the Th2-type immune response [37–39]. Thus, it is not surprising that either a combined vaccine or an inactivated FMD vaccine would be capable of eliciting ELISA antibodies and neutralizing antibody titers that were stronger than the cellular immune responses against FMDV serotypes O and Asia 1. However, antibody-mediated and cellular-mediated responses were stable and seemed to be higher than the cut-off level titers after 1 MPV until the conclusion of the experiment. Additionally, Barnett et al. [40] suggested that the VNT titers to FMDV among the animals with VNT titers for serotypes O and Asia 1 were higher than 2.068 and 2.252, respectively. Furthermore, they were capable of offering protection against FMD infection at a degree of probability of 95 %. According to the VNT titers obtained from the present study, it was implied that the cattle immunized with an FMDV-containing vaccine in this study provided the appropriate protective immune responses to protect against FMDV infection by determination of our derived titers. Considering the immune responses to the combined FMD-HS vaccine, no differences were observed when compared to the inactivated FMD vaccine. It was demonstrated that the compatibility of a combination of immunogens revealed no interference of immunogenicity between the FMD and HS antigens. It was further indicated that there was no antagonizing effect of rOmpH on the cattle immune response to the FMD vaccine [30]. These observations were similar to those of previous studies in which the FMD vaccine was combined or simultaneously administered with other vaccines such as vaccines administered for the bovine ephemeral fever [41], the rift valley fever [42], the combined rabies virus, *P. multocida* and *Clostridium chauvoei* antigens [43], and the rabies virus [44]. However, a synergistic effect has been reported with regard to the immunogenicity of both FMDV and various antigens [28, 30, 45, 46]. Altogether, these results support the contention that the incorporation of various vaccines along with the FMD vaccine could be successfully developed for practical use with no impact on the immune response. Moreover, the results of the Lymphocyte proliferation assay were consistent with those of previous studies in which the cellular response was not hampered in a comparison between the FMD antigen and another antigen [30, 47].

Previously, an inactivated HS vaccine was incorporated with the FMD vaccine in order to produce a combined vaccine [30–32]. Here, we have produced the rOmpH of the *P. multocida* strain M-1404 (B:2) and developed a novel combined FMD-HS vaccine. Our results revealed that two vaccine formulations containing rOmpH could elicit high antibody titers and cellular responses against the heat extract antigen of *P. multocida* throughout 8 months of the experiment. These results were in agreement with those of Prasannavadhana et al.[22] who reported that rOmpH is noteworthy for its immunogenicity, while Ataei et al. [48] reported that OmpH was not a strongly immunogenic protein. Moreover, these results were consistent with those of previous studies in which a rOmpH-based vaccine could strongly elicit efficient humoral and cell-mediated immune responses in animals [6, 21, 24]. Furthermore, our results revealed that the antibody titers against HS were higher in the combined FMD-HS vaccine groups than in the HS vaccine groups. This outcome was previously observed in ducks that were immunized with rOmpH combined with the duck enteritis virus (DEV) vaccine [49]. Considering the immune response against rOmpH, due to OmpH is a porin protein of *P. multocida*. It is known to be able to modulate the expression and release of IFN-gamma and IL-12. These substances are known to be involved in the selection of a Th1 immune response [10] resulting in induced high titers of specific antibodies and strong T cell proliferative responses, for which balanced Th1 and Th2 responses were observed against *P. multocida* in mice models [50]. Subsequently, the immune response would be synergized with predominant humoral responses from the FMD antigen. Taken together, the results indicate that rOmpH could serve as a potential protein antigen and could be combined with the FMD vaccine to protect against HS and FMD without antigenic competition.

## Conclusions

This study has provided a clearer understanding of the immune responses of cattle that had been immunized with a novel formulation of a combined vaccine consisting of inactivated FMD vaccine and rOmpH of *P. multocida* under field conditions. The findings are significantly helpful in demonstrating how a novel combined vaccine will behave in a group of animals under field conditions. Additionally, this could further reflect the true immunity status of the subjects. Therefore, these results can be useful for those individuals overseeing FMD vaccination monitoring in Thailand. To the best of our knowledge, this is the first study on the use of rOmpH incorporated with FMD vaccine to develop a combined vaccine against FMDV and HS. Notably, the immune response results are very promising. The preliminary study assessment under field conditions demonstrated that the combined FMD-HS vaccine can be safely administered and can achieve a degree of immunogenicity without any adverse events. Furthermore, it could provide high immune response and long-lasting immunity in immunized cattle under field conditions. Altogether, the data compiled in this study revealed that the combined vaccine could serve as an interesting alternative vaccine to protect cattle against FMD and HS with improved efficacy and safety when compared to the vaccines that are presently being used. However, in order to gain a comprehensive understanding of its potential, further studies would be needed that take into account the stability of the vaccine, the appropriate variety of its formulations, its adjuvants and its relevant degree of protectivity.

## Methods

### Animals and sample size

The sample size in this study was determined according to previous clinical trials involving a combination vaccine (significance level of 0.05 and power of 0.80) [43]. The minimum sample size required to conduct our study was 10 samples for 3 groups. The sample size calculation was done using G*power software (Version 3.1.9.2). Thirty healthy dairy cattle subjects (Holstein Friesians) that were 4–6 months old were used in this study. Dairy cattle were kept in a free-stall barn. All the cattle were acquired from farm members under the Mae Wang Dairy Cooperative, Mae Wang District, Chiang Mai Province, Thailand. The cattle were all screened for anti-*P. multocida* serovar B:2 antibody by indirect ELISA and anti-FMD by LPB-ELISA as has been previously described [1, 4, 51, 52]. Eventually, the dairy cattle were retained in the farm for milk production without culling or euthanasia at the end of the experiment.

### Vaccine preparation

#### Production of rOmpH protein and rOmpH-based HS vaccine

The rOmpH protein was produced according to the method described in a previous study [24]. Briefly, the *E. coli* strain M15 containing an expression pQE-30 vector (The QIAexpressionist™ Kit, QIAGEN, Hilden, Germany) inserted with the *ompH* gene of *P. multocida* strain M-1404 (serovar B:2) (pQE-30/*ompH*) was cultured in selective LB broth containing 100 µg/ml ampicillin and 25 µg/ml kanamycin (Sigma Aldrich, St. Louis, MO, U.S.A.). When OD 600 nm reached 0.5, the rOmpH protein was expressed by adding Isopropyl-β-D-thiogalactopyranoside (IPTG; Takara, Otsu, Japan) at a final concentration of 1 mM. The crude protein was collected and the rOmpH protein was subsequently purified using the electroelution method as has been previously described [24, 53]. The rOmpH concentration was measured using the BCA protein assay kit (Pierce®, Rockford, IL, U.S.A.) according to the manufacturer’s instructions. The rOmpH-based HS vaccine was formulated by the in-house mixing of rOmpH with the Montanide ISA 206 VG adjuvant (1:1 V/V, SEPPIC, Paris, France). A single dose (1 ml) of the HS vaccine contained 100 µg of purified rOmpH [6].

#### Bivalent inactivated FMD vaccine preparation

The commercial FMDV bivalent vaccine contained two strains of an inactivated FMDV including serotypes O/TAI/189/87 (10^7^ TCID_50_) and Asia 1/TAI/85 (10^7^ TCID_50_) formulated with an aluminum hydroxide gel adjuvant. It was manufactured by the Bureau of Veterinary Biologics, Department of Livestock Developments, Ministry of Agriculture and Cooperative, Pak Chong, Nakhon Ratchasima, Thailand (DLD, Thailand).

#### Generation of combined FMD-HS vaccine

The combined vaccine was prepared by mixing 100 µg of purified rOmpH with 2 ml of the commercial FMDV bivalent vaccine (DLD, Thailand). Briefly, the purified rOmpH protein was aseptically added into the FMDV vaccine bottle. The combined vaccine bottle was sealed tightly and then mixed by inverting the bottle. The combined FMD-HS vaccine was kept in 4 °C until using.

#### Experimental design

**Fig. 5:**
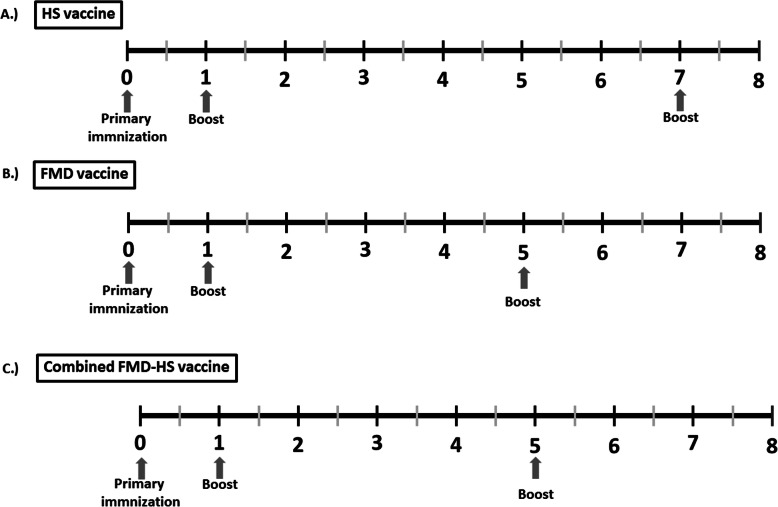
Timeline of experiments that involved cattle

Thirty cattle that were seronegative (the titers showed in the Results section) for FMD and HS were equally divided into 3 groups. The HS vaccine group was intramuscularly immunized (n = 10). The FMD vaccine group (n = 10) and combined FMD-HS vaccine group were subcutaneously (n = 10) immunized following the FMD vaccine program that was recommended by the DLD and the Mae Wang Dairy Cooperative.The administered vaccination program is shown in Fig. 5. Blood samples were collected before vaccine immunization and every month for 8 months. The blood samples were subjected to immune response analysis.

#### Determination of anti-rOmpH IgG antibody using indirect enzyme-linked immunosorbent assay (ELISA)

Indirect ELISA was performed according to the method previously described [51]. The *P. multocida* strain M-1404 (serovar B:2), which was kindly provided by Professor Dr. Takuo Sawada, Laboratory of Veterinary Microbiology, Nippon Veterinary and Life Science University, Tokyo, Japan, was used to prepare the heat extract antigen as a coating antigen [51]. Flat-bottomed 96-well plates (Nunc-Immuno Plate MaxiSorp, Intermed, Roskildes, Denmark) were coated with 100 µl/well of 160 µg/ml of the heat extract antigen diluted in the coating buffer (0.05 M carbonate bicarbonate buffer, pH 9.6). After being washed three times with washing buffer (0.05 % TWEEN 20 in phosphate-buffered saline (PBST)), the plates were blocked with 100 µl/well of blocking buffer (1 % skim milk in PBS, pH 7.2) and incubated for 1 h at 37 °C. After being washed three times with PBST, 100 µl of serum diluted with the blocking buffer (1:100) was added to each well and incubated for 1 h at 37 °C. After washing the plates with PBST, horseradish peroxidase-conjugated goat anti-bovine IgG (KPL, Gaithersburg, MD, U.S.A.) diluted with blocking buffer (1:2,000) was added and the plates were incubated for 1 h at 37 °C. The reaction was developed by adding 3,3',5,5'-tetramethylbenzidine (TMB; KPL). The color reaction was stopped by adding 50 µl of 2 M H_2_SO_4_. The absorbance was read at a wavelength of 450 nm.

#### Determination of anti-FMDV IgG antibody using liquid phase blocking ELISA (LPB-ELISA)

LPB-ELISA fraction was performed by the method previously described [1, 52]. ELISA plates were coated with 50 µl/well of rabbit antibody (anti-FMD virus antigens of serotypes O and Asia 1) in 96 well ELISA plates in duplicate. Meanwhile, a 50 µl of a duplicate, twofold series of each test serum is prepared, starting at 1:8 in U-bottomed multiwell plates (carrier plates). To each well, 50 µl of a constant dose of FMDV antigen (homologous to the rabbit antisera used to coat the plates) is added and the mixtures are incubated overnight at 4 °C. On the second day of the test, the ELISA microplates were washed three times with washing buffer (PBST). Then, a 50 µl of the mixture serum/antigen was transferred from the carrier microplate to the ELISA microplates. The plates were incubated at 37 °C with rotary shaking for 1 h. After then being washed three times with PBST, 50 µl of anti-FMDV type-specific guinea pig antibodies (1:1,000 diluted in PBST + 5 % skim milk) were added to each well and incubated on a rotary shaker at 37 °C for 1 h. The plate was washed three times with PBST. Then, 50 µl of the horseradish peroxidase-conjugated antibody (polyclonal rabbit anti-guinea pig IgG) (1:3,000 diluted in PBST + 5 % skim milk) was added to teach well and the plates were then incubated at 37 °C for 1 h. The plate was washed three times with PBST. A volume of 50 µl of TMB  (KPL) was added to each well. The plate was then incubated at room temperature for 15 min. Finally, 50 µl of stop solution (1 M H2SO4) was immediately added to all the wells. The absorbance was read using a microplate reader at 492 nm. The log10 antibody titers were expressed as the log10 of the reciprocal of the final dilution of serum giving 50 % of the mean OD value recorded in the absorbance of the control wells [1, 54]. The cut-off point was set to log10 titer equal to 1.60 [1].

#### FMDV virus neutralization test

The virus neutralization test was performed according to the method recommended by the World Organization for Animal Health manual [1]. Briefly, cattle sera samples were inactivated at 56 °C in a water bath for 30 min before being used. Subsequently, two-fold serial dilutions of the serum were mixed with purified FMDV (serotypes O/TAI/189/87 and Asia 1/TAI/85) suspension containing 100 TCID_50_ (50 % tissue culture infective dose) in flat-bottomed microtiter plate. The mixture was incubated at 37 °C in 5 % CO2 for 1 h. A 50 µl of the BHK-21 (ATCC® CCL-10™) (10^6^ cell/ml) grown on Dulbecco’s Modified Eagles Medium (DMEM, Gibco, Gaithersburg, MD, USA) (DMEM supplemented with 1 % antibiotics-antimycotic (Invitrogen), 10 % fetal calf serum (FCS, Invitrogen)) was added to each well. The microtiter plate was then incubated at 37 °C in 5 % CO2 atmosphere for 2–3 days. After 48 h. The plates are finally fixed with 10 % formol/saline and stained in 0.05 % methylene blue in 10 % formalin routinely on the third day. The plates are rinsed in tap water. Positive wells are seen to contain blue-stained cells sheets; the negative wells are empty. Titers are expressed as the final dilution of serum present in the serum/virus mixture where 50 % of wells are protected [55]. The cut-off point was set to log10 titer = 1.60 (titer dilution of 1:40) as seropositive [1].

#### Determination of cellular immune response

Peripheral blood mononuclear cell (PBMC) isolation was performed with minor modifications as has been previously described [6, 56]. Whole blood samples (10 ml) were collected from cattle in ethylene diamine tetraacetic acid (EDTA, BD Vacutainer, Plymouth, UK) tubes. Blood samples were diluted with sterilized PBS (pH 7.2) to a final volume of 15 ml and underlaid with 10 ml of Lymphoprep™ (STEMCELL Technologies, Vancouver, Canada). PBMCs were separated as a thin layer over the Lymphoprep by centrifugation at 400 × g for 30 min at 4 °C. PBMCs fractions were collected and the contaminating red blood cells were lysed by the 1× red blood cell lysis buffer for 5 min at 37 °C. PBMCs were then washed twice with RPMI 1640 by centrifugation at 700 × g for 7 min at 25 °C. Then, cell pellets were resuspended with 2 ml complete RPMI medium (RPMI 1640 medium supplemented with antibiotics-antimycotic (Invitrogen), 10 % fetal calf serum (FCS, Invitrogen) and 2.5 × 10^− 5^ M 2-Mercaptoethanol) before enumerating the number of cells. PBMCs at 2 × 10^5^ cells/well were stimulated with 5.0 µg/ml (final concentration) of heat extract antigen of *P. multocida* strains M-1404 or 10^5^ TCID_50_/2µl of purified FMDV antigens (serotypes O/TAI/189/87 and Asia 1/TAI/85) for HS or FMD testing, respectively. This step was performed in duplicate in 96-well plates. A final concentration of 10 µg/ml of ConA (ConcanavalinA, C-2010, Sigma) was used as a positive control. Plates were incubated for 48 h at 37 °C in an atmosphere containing 5 % CO_2_. The effect of the stimulated groups on the lymphocyte proliferative ability was measured using the 3-(4, 5-di-methylthiazolyl-2)-2, 5-diphenyltetrazolium bromide (MTT) assay. At the completion of 48 h of incubation, 10 µl of 12 mM MTT solution (Sigma-Aldrich) was added to each well. Three hours after incubation, 100 µl of SDS-HCl solution was added into each well and they were then incubated for a further 3 h at 37 °C. Bioassay response was quantified by reading the absorbance at 540 nm using an automatic plate reader (AccuReader). Results were expressed as SI and were calculated as SI = mean absorbance in stimulated wells/mean absorbance in non-stimulated wells.

### Statistical analysis

Antibody levels (HS and FMD) were analyzed using the R statistical software program (Version 3.2.2) [57] in order to determine the differences in mean between vaccine groups each month (month = 1, 2, …, 7 and 8).Sera from each cattle were collected monthly, thus data were correlated. Therefore, data were analyzed using a generalized linear mixed model (GLMM) to determine the overall effects of the vaccine type on the antibody level for the entire study period. The GLMM defined the vaccine group, time and interaction between vaccine group and time as fixed effects whereas an individual cattle was defined as a random effect. For GLMM model, we fit the models with different correlation structure including symmetry, autoregressive process of order 1 (AR1) and general correlation with no structure in order to find the best-fitted model. The model with structure that has the lowest Akaike information criteria (AIC) was concluded as the final model.

In terms of the post hoc analysis, linear contrasts were constructed to analyze the differences in mean values between the vaccine groups at each month using Tukey’s test. The significance level was set at α = 0.05.

Statistical analyses of the SI value between the vaccine alone groups and the combined vaccine groups were performed using a repeated-measures ANOVA test. The level of significance was recorded at *p* < 0.05.

## Data Availability

All relevant data in this study are available from the corresponding author upon reasonable request.

## Abbreviations

FMD: Foot and mouth disease
HS: Heamorhagic septicemia
OmpH: Outer membrane protein H
rOmpH: Recombinant outer membrane protein H
OIE: World organisation for animal health
DEV: Duck enteritis virus
FMDV: Foot and mouth disease virus
OD: Optical density
MPV: Month post-vaccination
ELISA: Enzyme-linked immunosorbent assay
LPB ELISA: Liquid phase blocking Enzyme-linked immunosorbent assay
VNT: Viral neutralization test
H_2_SO_4_: Sulfuric acid
TMB: 3,3',5,5'-Tetramethylbenzidine
SI: Stimulate index
DLD: Department of livestock development
FMD Vaccine: Foot and mouth disease vaccine
HS Vaccine: Heamorhagic septicemia vaccine
Combined FMD-HS Vaccine: Combination vaccine containing inactivated foot and mouth disease virus and recombinant OmpH of *P. multocida*
LPA: Lymphocyte proliferation assay
DMEM: Dulbecco’s modified eagles medium
PBMCs: Peripheral blood mononuclear cells
PBS: Phosphate-buffered saline
PBST: Phosphate-buffered saline containing 0.05 % Tween® 20
TCID_50_: 50 % tissue culture infective dose
CPE: Cytopathic effect
RBC: Red blood cell
FCS: Fetal calf serum
MTT assay: 3-(4,5-di-methylthiazolyl-2)-2, 5-diphenyltetrazolium bromide assay
SDS-HCl: Sodium dodecyl sulfate- hydrochloric acid
